# “I Know How to Advocate”: Parents’ Experiences in Advocating for Children and Youth Diagnosed With Autism Spectrum Disorder

**DOI:** 10.1177/11786329221078803

**Published:** 2022-02-24

**Authors:** Joanne Smith-Young, Roger Chafe, Rick Audas, Diana L Gustafson

**Affiliations:** 1 Division of Clinical Epidemiology, Faculty of Medicine, Memorial University, St. John’s, NL, Canada; 2 Division of Pediatrics, Faculty of Medicine, Memorial University, St. John’s, NL, Canada; 3 Division of Community Health and Humanities, Faculty of Medicine, Memorial University, St. John’s, NL, Canada

**Keywords:** Child advocacy, autism spectrum disorder, parents, qualitative research

## Abstract

**Background:**

Parental advocacy is a dynamic process that changes depending on the circumstances and needs of the child and parent. Communication deficits related to an Autism Spectrum Disorder (ASD) diagnosis often necessitate parental advocacy. This study describes how parents and caregivers of children and youth diagnosed with ASD engage in parental advocacy, the challenges they encounter and the advocacy skills they develop.

**Method:**

We used descriptive exploratory methodology informed by reflexive thematic analysis. The aim of the study was to explore advocacy in parents and caregivers of children and youth diagnosed with ASD.

**Results:**

We conducted in-depth, semi-structured interviews with 15 parents of children and youth with an ASD diagnosis living in 4 provinces of Atlantic Canada. The pathway in parents’ advocacy journey included: (1) Expressing concerns; (2) Seeking help, assessment, and diagnosis; (3) Acquiring services; (4) Removing barriers; and (5) Developing advocacy skills.

**Conclusions:**

Our findings illustrate the process of parental advocacy, skill development, and the barriers parents encounter in advocating for their children with ASD. Future research might explore how health professionals can support parents’ advocacy efforts.

## Background

Autism spectrum disorder (ASD) is a chronic neurodevelopmental disorder, with patients often showing symptoms before the age of 4.^1^ There is no blood or genetic test to diagnose the disorder. Clinicians usually rely on observations, medical histories, and a variety of diagnostic and assessment tools to determine whether an individual has ASD.^2^ Common features of ASD include atypical eye contact, hyperactivity, and repetitive body movements such as rocking or hand-flapping, with patients having a wide range in the severity of their symptoms.^2^ Individuals with ASD may also have mild to moderate intellectual disabilities, but again the severity of impairment varies widely from patient to patient.^3^ One of the defining features of ASD is a significant deficit in social, communication, and interaction skills.^1^ For example, children with ASD display lack of normal back-and-forth conversations, failure to initiate or respond to social interactions, and difficulties maintaining and understanding relationships.^1^ These social deficits can increase the demands on parents to advocate directly on behalf of their children.

Children with ASD require access to a range of health and educational services and supports. Parents are often the main advocates for their children across these various programs.^4^ Parents need to report their perceptions of their children’s health conditions to medical and other professionals to receive a diagnosis and access appropriate services. Children with ASD often have additional co-morbidities, such as Attention Deficit Hyperactivity Disorder, seizure disorders, anxiety, and gastro-intestinal disorders. Co-morbidities can make accessing diagnostic and treatment services more difficult.^1^

Parents of children with ASD are faced with many challenges. A Canadian study showed that having a child with ASD resulted in parents experiencing a sense of isolation because of a perceived lack of understanding from society and unsupportive system.^5^ Results showed experts and professionals lacked the necessary knowledge and expertise in dealing with the needs of families.^5^ Parents experience a sense of loss both for themselves and other family members.^5^ Having a child diagnosed with ASD has direct and persistent impacts on the family, including financial, social, and marital and sibling relationships.^2^ Marital difficulties can lead to family breakdown and divorce.^6^ Challenges in caring for a child with ASD can result in higher levels of stress that can affect parents’ mental health and well-being.^6^

Parents are sometimes reluctant to advocate in the school system for their child with ASD because they lack essential knowledge of the education process; thus, they often request support from a special education advocate when it is available to them.^7^ When disagreements or disputes occur between the parents and the school administrators about a student, mediation is an option. Mediation is a voluntary process aimed at establishing agreement between parents and administrators.^8^ If mediation is unsuccessful, then a due process hearing is a legal proceeding that attempts to resolve the dispute.^8^ In the US, parents of children with ASD are more ten times more likely to pursue legal advocacy when compared to comparator groups in other disabilities categories. Some scholars argue that this is “likely due in part to the school systems’ limited success in effectively addressing this complex disability (p. 98).”^9^

Advocacy is a dynamic process that changes depending on the circumstances and needs of the child and parent. Parents are considered natural advocates because of their assumed commitment to the well-being of their child.^4^ Many parents view advocacy as a moral obligation or expectation.^10^ Advocacy can be an active coping strategy for parents of children with disabilities.^11^ However, not all parents and guardians are in a position to effectively advocate for a child with ASD. There is debate about whether parents sometimes intervene too much on their children’s behalf.^12^

Researchers describe advocacy in the ASD literature as “any action taken by a parent on behalf of their child or other children with ASD to ensure adequate support, proper level of care, and basic human rights (p. 74).”^11^ Advocacy skills for parents of children with ASD may include understanding ASD, using clear and effective communication, being organized, and managing difficult situations when they arise.^13^ Several contextual factors may increase or decrease parental advocacy over time. Some of these factors include financial status, education and skills, time commitment, severity of the child’s condition and age of diagnosis.^14^

### Financial status

Economic status and family income can affect parents’ level of empowerment and their ability to advocate for their children with ASD.^14,15^ High-income parents are better able to meet their child’s needs for services and support by paying out-of-pocket.^11^ Parents are often willing to pay significant sums of money to help their children often causing financial hardship.^16^ Parents report feeling “blessed” when they have both the financial status, education, and skills to “fight” for their children diagnosed with ASD.^17^

### Education and skills

Parental advocacy is an ongoing and multifaceted communicative process that includes staying informed and educated.^18^ To advocate effectively, parents need to educate themselves to gain an understanding of the relevant social, economic, and political environments and become familiar with service delivery, legislation, and budgetary issues.^11^ Parents are often challenged in their advocacy efforts because they have difficulties in navigating the system.^19^ It seems parents become more comfortable advocating for their child as they become more educated and skilled with their child’s diagnosis.^20^ Parents are more likely to engage in advocacy efforts when they develop effective self-efficacy skills.^10^ In fact, parents who achieve a mastery of skills in their ability to advocate feel empowered to act on their child’s behalf.^14^

### Time commitment

Advocacy and caregiving can be a full-time activity for parents with 1 parent often having to give up work to advocate.^16^ Meetings scheduled at short notice make it difficult for working parents or parents involved in childcare and other responsibilities to attend. The time parents spend traveling from rural to urban centers to acquire ASD services is another challenge to parental advocacy efforts.^15^ Time and resources impact parents’ ability to engage in advocacy.^19^ Parents tend to become more involved in broader systemic advocacy after their children are older or their conditions improve when they have more time to do so.^17^

### Severity of condition and age of diagnosis

The severity of the child’s ASD diagnosis affects parents’ conceptualization of autism in ways that influence their ability to advocate.^15,17,21^ Parental advocacy also depends on the age of the child with ASD.^17^

This paper addresses a gap in the existing literature about the circumstances surrounding parental advocacy for children and youth with ASD. This descriptive exploratory study examines the question: “When, how, and why do parents of children and youth with ASD engage in parental advocacy and what barriers, if any, do they encounter?” Improving our understanding of parents’ experiences of advocacy may help other parents of children and youth with ASD be effective advocates. Results from this study will also point to ways that service providers can promote parental advocacy by developing collaborative and constructive partnerships with parents and providing timely and appropriate services over the life course of the condition.

## Method

### Design and data collection

This paper draws on a data set that was part of the Atlantic Canada Children’s Effective Service Strategies in Mental Health (ACCESS-MH) project conducted in Newfoundland and Labrador, Prince Edward Island, New Brunswick and Nova Scotia. One of the goals of the ACCESS-MH project was to explore the journeys of parents with a child or youth diagnosed with a developmental disorder, including ASD.

We used qualitative descriptive exploratory methodology^22-25^ to explore and describe parents’ experiences in raising a child diagnosed with ASD in relation to advocacy. Purposeful sampling was used to recruit participants for the study. We developed a poster to recruit participants through various ASD advocacy groups within the 4 Atlantic provinces who distributed the poster for us and endorsed our research. Interview data were collected from parents who identified as having a child or youth diagnosed with ASD. Data were subject to reflexive thematic analysis.^26^ Two research assistants under the supervision of the senior researchers from the project team conducted face-to-face interviews with parents in a private office setting. The interviewers were male and female university students. None had a child with ASD. Field notes were taken during each interview. Memorial University’s Human Investigation Committee provided ethics approval for this study. Each consenting participant provided written informed consent prior to being interviewed and audio-recorded.

During hour-long in-depth, semi-structured interviews, parents were asked to describe their journey of parenting a child with ASD. They discussed their child’s concerning behaviors prior to receiving a diagnosis, how they obtained services and supports for their children, and any barriers faced in their advocacy efforts. Parents also offered recommendations for helping other parents who may be going through the same type of journey.

Interviews were transcribed verbatim. All identifiers were removed and numeric codes were used (eg, P-1, P-2, etc.) to identify participants to assure patient confidentiality.

### Data management and analysis

The data analysis process was informed by Braun and Clarke’s 6-phase process for conducting a thematic analysis, which includes 6 steps: familiarizing yourself with the data, generating initial codes, searching for themes, reviewing themes, and defining and naming themes.^26^ In order to achieve data familiarity, the first author (JSY) engaged in repeated reading of the transcripts helping to move the analysis beyond a focus on the most obvious meanings. During initial readings of the transcripts, parental advocacy inductively emerged as a prominent aspect of support seeking journeys, even though the original interview protocol did not initially focus on this topic. Once parental advocacy was defined as a relevant concept, it was used as a sensitizing concept in data analysis.^27,28^ Data collection ceased at the point where there was enough data to “build a comprehensive and convincing theory (p. 148)”^29^ in the sense of theorizing based on data collection.

Three authors (JSY, RC, and RA) corresponded several times throughout the data analysis process to identify preliminary codes and themes, and discuss patterns of behaviors. All data relevant to the research question were coded. This process identified the first patterns in the data by grouping similar data segments. Similar codes were then clustered together to create a visual map of key patterns in the data that contained overarching themes. The first author (JSY) kept a log of thoughts and reflections on preliminary and developing codes during the thematic analysis process, as recommended by Braun and Clarke.^26^ All authors (JSY, RC, RA, and DG) reviewed the themes to ensure they fit with the coded data and the overall data set. Discrepancies in coding were discussed until consensus was achieved. We then selected quotations deemed particularly representative of the themes agreed upon by all authors (JSY, RC, RA, and DG).

To ensure a rigorous study design, we used the verification strategies outlined by Morse^30^: methodological coherence, appropriateness of sample, concurrent data collection and analysis, and theoretical thinking and theory development. Methodological coherence includes attention to congruence between the research question and the methodological components.^30^ The topic under study is well suited to qualitative descriptive exploratory methodology because our goal was to understand parents’ experiences of advocacy as described in parents’ own words. Our data were collected from parents of children with ASD who had engaged in advocacy and were knowledgeable about the phenomenon under study making them an appropriate sample.^30^ We engaged in concurrent data collection and analysis. This iterative process required that we moved back and forth between what was known and what further knowledge was needed.^30^ We were open and flexible to ideas and consulted with each other as the process unfolded. This fulfilled the final strategy of in an emergent process of theoretical thinking and theory development.

## Results

Interview data were collected from 15 parents of children and youth diagnosed with ASD living in Atlantic Canada. Table 1 shows the participant demographics.

**Table 1.** Participant demographics.

Participant demographics		Number of participants
Province	Prince Edward Island	5
	New Brunswick	1
	Nova Scotia	3
	Newfoundland and Labrador	5
	Did not respond	1
Community	Urban	7
	Remote	6
	Did not respond	2
Gender of parents	Male	1
	Female	14
Age categories	31-40	3
	41-50	8
	51-60	4
Marital status	Married/Common law	11
	Divorced/Separated/Widowed	4
Education level	High school	3
	College diploma/some university	1
	Undergraduate degree	4
	Graduate degree	7
Employment status	Employed full time	13
	Unemployed	2
Gender of child	Male	12
	Female	3
Age of diagnosis of ASD	2-5 y	8
	6-9 y	4
	10-14 y	3
Child’s diagnosis	ASD	8
	ASD and other disorders including anxiety, depression, eating disorder, sensory integration disorder, obsessive compulsive disorder, oppositional defiant disorder, attention deficit hyperactivity disorder, conduct disorder)	7
Total yearly household income	$20 000 to less than $30 000	2
	$60 000 to less than $70 000	1
	$70 000 to less than $80 000	2
	$80 000 to less than $90 000	1
	Over $90 000	6
	Did not respond	3

One parent [P-14] did not wish to be quoted directly. We did not include any direct quotes from this participant, but their data were included in our analysis.

Advocacy was a key feature in parents’ descriptions of their journey with a child diagnosed with ASD. Parents regarded advocacy as a dynamic activity that changed depending on the circumstances and their needs or that of their children. Advocacy efforts required skill development and confronting barriers to advocacy that was central to their advocacy work. Thematic analysis revealed that the pathway in parents’ advocacy journey included 3 main themes and various sub-themes, as reflected in Table 2: (1) Engagement in Parental Advocacy (expressing concerns, seeking help, assessment, and diagnosis, acquiring services, and raising awareness); (2) Challenges or Barriers (time commitments, financial challenges, lack of knowledge and support, lack of service availability, system bureaucracies, and perceived stigma); and (3) Development of Advocacy Skills (active involvement/engagement and self-learning strategies).

**Table 2.** Primary and secondary themes in parents’ advocacy journey.

Engagement in parental advocacy	Challenges or barriers	Development of advocacy skills
• Expressing concerns • Seeking help, assessment, and diagnosis • Acquiring services • Raising awareness	• Time commitments • Financial challenges • Lack of knowledge and support • Lack of service availability • System bureaucracies • Perceived stigma	• Active involvement/engagement • Self-learning strategies

### Theme 1: Engagement in parental advocacy

#### Expressing concerns

Advocacy efforts began when parents first identified concerns about their child and started to connect those concerns with suspected ASD behaviors. Some parents recalled noticing signs during infancy. “*I noticed probably within the first three months that my child really didn’t really love to be snuggled*” (P-4). They identified difficulties with language skills, demonstrating ritualistic, repetitive and restricted behaviors, reacting to sounds and lights, having tantrums, or becoming overly upset when routines were changed. “*He really obsessed over those routines. If we didn’t do them that way, he would be very upset, and he wouldn’t settle down*” (P-7). Parents described feelings of uncertainty, fear, and guilt during this time. Some parents expressed disbelief when others expressed concerns about their child’s behaviors that they hadn’t noticed themselves.

I never saw it myself. . .I just assumed every 3-year-old knows what a Pachycephalosaurus [type of dinosaur] is and it was okay he was reading novels in kindergarten. . .he was just being a little inappropriate and struggling to make friends. (P-1)

Demonstrated behaviors such as separation anxiety and other forms of anxiety would trigger some parents’ concerns. Feelings of uncertainty and concern for their child’s condition motivated parents to take action in seeking help, assessment, and diagnosis for their child.

#### Seeking help, assessment, and diagnosis

Parents first started seeking help within the healthcare system. They wanted to find out what was the reason for their child’s symptoms and challenging behaviors. Parents reported on their encounters with health care providers in seeking help. Parents demonstrated strong emotive reactions during this time. At times parents’ advocacy efforts were described as combative with health professionals who took a “wait and see” approach that led them to look elsewhere for answers.

He [pediatrician] was like, well, wait and see over the next – because he was so young – over the next 3 to 6 months and I was like, ‘Are you. . .kidding me? You just told me you think my kid might have autism, I’m not doing the wait and see approach’. . .I went home and called [another pediatrician] and she booked us in like two weeks later - so we saw her within 2 weeks and she did the Autism Diagnostic Observation Schedule [ADOS] on the spot. . .she did the full testing at the initial visit and gave us the diagnosis. . .for sure we hopped the queue because until you get a diagnosis you don’t have access to autism intervention. . . if I wasn’t aggressive, if I hadn’t called them, it would probably have taken another 6 to 12 months I would say. (P-10)

This “wait and see” approach that health providers used resulted in some parents having to go to extremes to demonstrate their concerns. The parent of a three-year-old believed her concerns were being ignored and reacted this way.

I knew a lot of his anxiety triggers so I kind of provoked him. He had a complete meltdown at the developmental pediatrician’s office. She felt at that time it was a good time to have him assessed. So, he was assessed at 3 years, 3 months and found to be on the spectrum. . .you’re relieved but you’re angry that it took that long. (P-12)

Parents advocated for their children by searching for a “good fit” between the health care providers and their child that often resulted in paying privately for what they considered better care services.

Parents articulated a genuine appreciation for those who provide supports. “*Some of my support is my pastor at [church]*” (P-5). Parents appreciated school administrators who were accessible, supportive, and addressed their concerns. “*The amount of support that I’ve had from the School Board here is unbelievable.*” (P-9)

He’s had a wonderful school to be able to adjust to his needs – have regular meetings at the beginning of the year. . .we meet partway through the year to talk about how things are going. These meetings have like 6 to 8 people in them – his teacher’s there, the resource teacher’s there, the vice principal’s there, the autism consultant’s there, the guidance counsellor’s there, [husband] and I are there. So, we’re communicating all the time with each other. . .you develop a real rapport with them and a trust relationship with them and that’s important. (P-3)

Parents expressed appreciation about flexibility in the workplace that enhanced their ability to advocate.

I’m so lucky I work here. My manager is so sympathetic and allows me to have the flexibility that I need to ensure that I can do what I need to do with him. So, if I need to come in late then I just work an extra 15 minutes. If I need to take a day off and I don’t have any time left, I’ll just work it on the weekend. My schedule has been pretty flexible. (P-13)

Once parents received a formal diagnosis of ASD for their child, the next step was to acquire the necessary services that led them to the next phase of advocacy in acquiring ASD-related services and supports for their child.

#### Acquiring services

Attempts at acquiring services for their child was especially challenging for parents. Parents described a sense of urgency and time pressure because they had learned early on that intervention was important for a child diagnosed with ASD to have the best outcome possible.

You feel like you’re in a race. At the time he was 3 years, 3 months and services would end at 6 [years of age] so we were like we’ve lost – we had suspicions since he’s 18 months, we’ve lost over a year and a half. We’ve only got a year and a half left – like you feel like you’re constantly having to run. . .you’re trying to get your ducks in a row like, okay, I’m going to put an ad out [for a behavioral support worker]. (P-12)

Some health professionals offered services and supports for parents as they waited for an official diagnosis. “*She [child psychiatrist] said, ‘I’d like to provisionally diagnose him with ASD.’ They started dipping into different things they could help us with even though he hadn’t been formally diagnosed. . .there was a wait time for diagnosis*” (P-8).

Parents who had the financial means to acquire ASD services for their children reported feeling “lucky” (P-10). Parents who could afford it provided art, music, aquatic, and equestrian therapies in addition to Applied Behavioral Analysis (ABA) therapy. ABA therapy is the practice of applying the psychological principles of learning theory in a systematic way to modify behavior. The practice is used most extensively in special education and the treatment of ASD.^31^

He’s been super lucky. . .40 hours of therapy. . .we’ve hired privately. . .a speech pathologist that we hired privately and he gets private lessons for kids on the spectrum for swimming and he’s in a therapeutic riding program. . .we have basically unlimited educational and financial resources between us so we’ve been able to do all the things he needs and we’ve been able to buy all the supplies that the therapists need. . .we’ve been very lucky, for sure. (P-10)

Gaining knowledge about ASD increased parents’ confidence in their ability to advocate for services.

He [psychologist] makes an incredible report for the parents. . .it’s books you can read, organizations you can contact, government programs that might be able to help, suggestions of social skills development. . .so that became our map because we didn’t have anything else to go by. . .one of the things that was suggested was getting in contact with the Autism Society and so we did that and that was another great resource. (P-3)

For parents of school-age children and youth, securing an official ASD diagnosis for their child meant they were able to acquire additional educational supports.

We got an increase of services actually in the school because now he had an actual diagnosis – it wasn’t just a learning disability. So because of that like the doors opened. Like we managed to get the autism support specialist and the consultant that works in the schools to work with [child] or observe him and things like that and the team meetings got bigger in terms of people around the table, and they got more frequent. (P-2)

Parents described their advocacy efforts as being watchful and diligent to ensure the system met their child’s needs. Once again, parents displayed strong emotions attempting to ensure supports and services for their children. Parents described how they planned aggressive tactics at times to ensure their child’s needs and rights to services were met.

When he was in school, I had to fight to get some funding from the Autism Support Program with the Department of Education and I really had to demolish doors. . .their guidelines were very, very fuzzy and the policies were like non-extent. . .it was terrible and so I had to fight, and I had to pick up the phone and go straight to the director of the program. (P-2)

#### Raising awareness

Parents wanted to raise awareness that it can be difficult to recognize signs of ASD because it did not always present itself in a typical manner. They talked about ASD being an “*invisible*” (P-1) condition that made it even more challenging to recognize. Parents also wanted to dispel myths about ASD and once again described strong emotional reactions during this time.

Not all autism presents itself in the typical way. Neither one of my kids flapped their hands or did any of the typical autistic stuff. It was all throwing food, blinking their eyes, fluttering and stuff like that. They need more awareness or at least don’t be dismissive. . .it needs to be done earlier because I think of the months, the years that were wasted. . .I’m on the top of a mountain screaming my guts out and no one’s listening. . .no one can hear me, and someone needs to help this child. (P-11)

Parents talked about the emotional aspect of raising a child with ASD and changing public perceptions about the condition.

You do go through a depression, and you mourn not only their lives but your own. You’re not going to have grandchildren. You’re not going to have this. . .It’s something to overcome at times but for the most part it’s been pretty beneficial. His memory is amazing. His abilities are incredible. (P-1)

Parents were seeing possibilities for their children if supportive services were made available to them.

He’s a technology wizard. He can take apart and put together a computer. He reinstalled the operating system on my home computer without my knowledge when he was 8 or 9. . .He said, “I’m a great fixer mom, I’m going to be an IT guy”. . .I don’t know if he’ll ever be independent. The possibility is there if he can have the services to support him. (P-13)

Central to parental advocacy work was removing barriers to challenges faced in their advocacy efforts and developing advocacy skills.

### Theme 2: Removing challenges or barriers

Parents described 5 challenges or barriers in their advocacy efforts and offered some solutions to the challenges faced. Barriers included: (1) time commitments required related to parenting a child with ASD; (2) financial challenges; (3) lack of knowledge and support from health care providers, other professionals, and family members; (4) lack of service availability and system bureaucracies; and (5) perceived stigma regarding an ASD diagnosis.

#### Time commitments

Parents described the time commitments involved in parenting a child with ASD. Time spent traveling and coordinating appointments in the health care system and education system was often challenging and time-consuming. Parents were required to balance work and other family members’ needs. Some parents reported they had quit work in order to have more time to advocate and care for their child with ASD.

Time spent coordinating services and supports for their child with ASD and seeking out opportunities that could aide in their child’s development such as, meeting with teachers and school administrators, as well as arranging recreational activities that would benefit their children. They mentioned the time commitment involved in advocacy work.

Advocacy things are often run by parents and parents with a child with special needs don’t have a lot of time to publicize and do the kinds of things like that – you don’t have time to go to the annual general meeting when there’s no babysitting and you can’t hand your kid off to anybody. (P-10)

#### Financial challenges

Parents confronted financial barriers in their advocacy efforts and attempts at acquiring services for their child. “*It’s about $1,200 to pay for the assessment to get it quicker. . .we couldn’t really afford it*” (P-8). Those who could afford to pay for services felt lucky but other parents talked about feeling “emotionally and physically-drained” in their financial struggles to pay for services.

We’re going into debt. . .My father had offered his house for sale to fund the visit [to specialists in another country] and any treatment that we needed. . .Once a semester I have to dish out $75 for social thinking. Once a year I dish out $400 for horse camp. Art therapy is $20 a pop. . .Parents are physically, mentally and financially drained. . .everybody is in debt . . . the average family is 25 to 100 K in debt because of autism. . .we’ve refinanced our house twice because of autism. I’m not ashamed to say that. . .it’s the middle-class people who are struggling financially. . .they don’t care if I’m paying off student loans. They don’t care if we’re putting food in my mother-in-law’s fridge. (P-11)

Parents made a decision to wait for public healthcare services or seek out private healthcare services to get their child assessed more quickly. For instance, participants who could afford it reported paying privately for psychoeducational assessments as means of achieving a formal diagnosis to achieve needed services for their children.

Nothing’s official until the doctor puts the approval. . .once we know what’s happening and where we’re at with it and the doctor starts helping us – we can make use of the programs with the government – disability tax credit for the kids so that it’s a little easier to help with his upbringing, do different things and working with different tools and different programs. (P-5)

#### Lack of knowledge and support

Parents spoke about the lack of information about services and supports available to them for their children, “*The information is not there. . .there are little potholes of help but there’s nobody there who’s really put all this together*” (P-4). Parents described interactions with service providers as either positive or negative.

Negative interactions created a sense of distrust that demonstrated some negative emotional responses in their advocacy efforts.

The first psychiatrist we went to see ended up being an absolute disaster. . .I thought my husband was actually going to hit him. He was getting so mad he was just shaking because he was becoming verbally abusive to me. . .And he started just putting his finger in my face like this and shouting at me and our child is sitting there witnessing this. And so, we left and we walked out the door and we kind of stopped and we said, “Okay, so how do we get another psychiatrist because we’re never walking in there again.” (P-3)

Parents felt unsupported when they shared with family and friends their concerns about their child. Other parents talked about not knowing where to turn to look for support. “*I’m not a doctor! I don’t know what to do! - I’m just a parent who’s trying to get help for their daughter*” (P-4).

Parents were gaining knowledge about why it was important to receive a formal diagnosis in order for them to obtain necessary ASD early intervention programs for their children. However, they confronted barriers that included wait times and dismissive health care workers that were taking a “watch and wait” approach. Parents with knowledge of child development had some insight into ASD symptoms, yet some parents felt health care providers were dismissing their concerns.

I have a minor in psychology, so I was familiar with child development, and I just noticed he wasn’t hitting his milestones and seeing our General Practitioner you know, it’s the typical story – boys don’t develop as quickly as girls. . .a wait and see basis. (P-12)

Parents perceived challenges related to hierarchical struggles in their encounters with health professionals.

He was the type of doctor who was patronizing you like he thought that everybody was like at this intellectual level and then he was here – it was terrible and he would not be open-minded and he was just stuck in his run and there was no way to discuss it in a very free way. (P-2)

Parents were learning about ABA services that were available for children with ASD, which can support developing relevant skills and behaviors. Parents were aware of the importance of early intervention and sought out ABA services that were available to them. However, parents felt unsupported and challenged in their efforts to find suitable service providers that were considered compatible with their child’s individual personality. One participant decided to “double the salary and pay out of pocket” for a home therapist who they considered had a good rapport with their child.

The parents are left to advertise, to find, to interview, to know what to look for, how to pay them. . .we went 7 months without an ABA therapist because not only could we not find people to apply, let alone hire someone. (P-11)

#### Lack of service availability and system bureaucracies

Parents living in rural or remote areas of the province faced challenges of limited access to services that were only available in urban centers requiring them to travel long distances to seek help for their child

The local hospital had very limited resources where we live. We live in a small town of 500 and [location of county] itself isn’t that big. . .we had to just kind of wait until we could see somebody and help us, like two and a half hours away. (P-7)

Parents wanted to educate others and learn from their experiences in confronting barriers in navigating the system searching for ASD diagnostic services and supports.

If you don’t feel something’s right and you’re getting ready to get pushed out the door, if you need to be a little bit strong in your advocacy, be stronger. . .I’m my child’s expert. . .I’m the one that’s with him every day 24/7. I’ll take their advice if I think it’s what’s best and it fits for him. (P-12)

#### Perceived stigma

Parents talked about instances when they felt blamed for their child’s inappropriate behaviors displayed in public and described they were “*made to feel like it was our fault somehow*.” (P-3) Parents expressed opinions about disclosing their child’s ASD diagnosis. Some thought it best to be open about it yet others felt it best to keep it to themselves because they feared stigmatization.

People look at the disability before they look at the person. . .I never ever wanted his diagnosis to be a crutch. I always wanted to see the potential. . .we kept it a secret because we wanted people to treat him typically (P-12).

Parents talked about the myths surrounding ASD as being a barrier.

I didn’t think my child could be like sweet and friendly and have autism - the myths are certainly alive and well and the thing about no empathy and stuff like that – you know people [with ASD] can talk. . .the second barrier would be the myths about autism (P-10).

As parents faced various challenges in the process of advocacy, they were also learning to develop advocacy skills that would help them overcome some of the confronted barriers.

### Theme 3: Development of advocacy skills

Parents identified several skills in advocating for their child that included being actively involved and engaging with health care providers and other professionals as well as self-learning strategies.

#### Active involvement

Parents became actively involved with care providers as they gained knowledge and began to identify signs of ASD in their children.

He was 5 [years old]. . .he saw a pediatrician and immediately after a 20-minute interview, “He’s got ADHD” I went home and I kind of researched and I said, “No, this is not my kid. This is not right.” I can see the parallels and I can understand but it felt wrong. . .[later the child was diagnosed with ASD after the [general practitioner] GP put them in touch with a psychologist]. . .she said it really is Asperger’s and I was like the first paragraph of the book that I picked, and I was like there’s my kid (P-1).

Parents described a wide range of behaviors including some negative emotional reactions in their advocacy efforts to obtain supports and services for their children. “*I’m very proactive in seeking help [for my child]. . .where there’s a will, there’s always a way*” (P-6).

I’m a very strong advocate and I’ve kicked doors, but I had to work really hard. . .I’ve been yelling and screaming on the top of my lungs sometimes to get him to where he is. . .that’s basically how we’ve navigated through the system. . .My husband and I also did a presentation. . .talked about our experience and how he [son] fell through the cracks many, many, many – too many times (P-2).

Parents spoke about educating teachers and students in their child’s school about ASD in an effort to gain understanding and support. “*I was actively involved with the principal. I was involved with the teachers. I was in the classroom*” (P-6).

#### Self-learning strategies

Parents were learning what skills were important in being an effective advocate. They talked about the importance of being realistic and self-aware in their advocacy efforts.

I can be an advocate but I’m not somebody who’s going to the media and complaining and bitching. I’m just not doing it. Advocate for your child when you need to advocate for your child but be realistic (P-13).

Parents reflected on ASD journey and the need for support for families. “*I think there needs to be more support for the parents. . .there needs to be more family support*” (P-15). Parents self-reflected on their own experiences with advocacy and talked about what other parents needed to support them in their journey of advocating for a child with ASD.

I am an advocate now working with people who don’t have the voice, don’t know how to use it or can’t figure out what to do or just accept what’s happening. . .we have a group on Facebook. . .It’s made me probably stronger than I’ve ever been before. . .I had no choice. I never want anybody to have to go through what we did. I feel in each community there should be a resource centre for families. . .better supports (P-7).

A parent described how their advocacy experiences had helped them to notice signs and symptoms in a younger sibling and how they felt better prepared to obtain supports and services for their second child.

This youngster [a younger sibling] came out into the living room one day, looked up out of the corner of his eye and started fluttering his eyelids. Me and [husband] looked at each other and said he has it too. . .Eighteen months just like that he fluttered his eye lids. . .we phoned Dr. [name]. Get the speech on the wait list, get the OT on the waitlist. Put him on the wait list. We were just from day one - we know the routine now so here give me that form, give me that form - I have to fill it out and get him on the waitlist (P-11).

Once parents gained the knowledge and skills to advocate effectively, they felt more confident in their ability to further their advocacy efforts. Parents described how it was important to raise awareness about ASD and educate others so they could benefit from their lived experiences.

I have enough education and drive and knowledge to have continued to push for my son. . .every bit of support that we got I had to fight for, pretty much tooth and nail fight for and lot of other parents don’t have the education or the energy to do that. . .people shouldn’t have to fight so hard (P-9).

**Figure 1.**
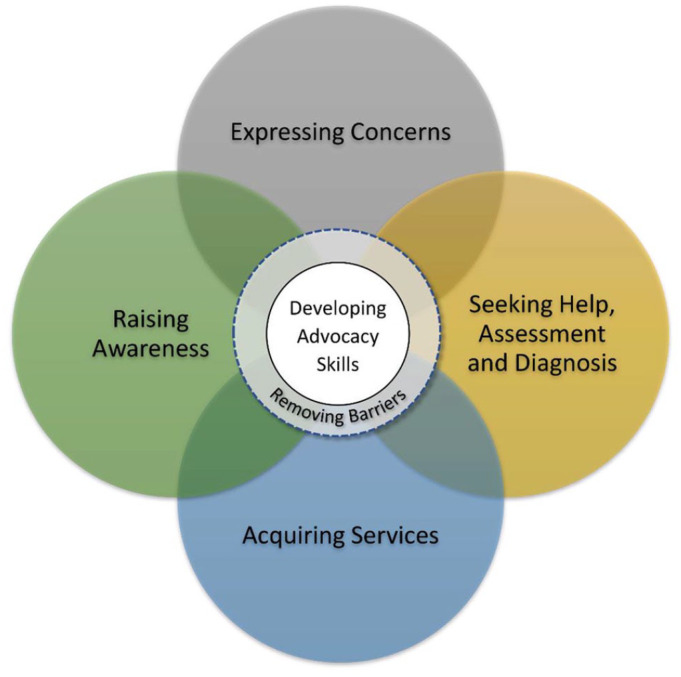
Pathway in parents’ advocacy journey with children and youth diagnosed with ASD.

Figure 1 is a thematic representation of the pathway in parents’ advocacy journey with children and youth diagnosed with ASD. Each of the large circles represent the pathway to engaging in parental advocacy and inform the removal of barriers and challenges to advocacy. Developing advocacy skills is central to this process.

## Discussion

The purpose of this study was to explore parental advocacy efforts in the journey of parenting a child with ASD. We offer a unique perspective by offering a thematic representation of the pathway in parents’ advocacy journey with children and youth diagnosed with ASD. This illustrates when, how, and why parents advocate on behalf of their children as they navigate barriers and supports in medical, educational, and social contexts of their child’s environment. The study findings reveal the barriers faced and the strategies used to overcome some of these challenges in the process of advocating for their children with ASD. We also describe the advocacy skills parents develop to support them as they continue their journey of advocacy throughout the life course of their child’s condition.

We found parental advocacy work to be dynamic as parents face uncertainty, seek help, acquire services, and promote awareness. Through various challenges and uncertainties faced, they continued their advocacy efforts to acquire services and supports for their children. Parents used their roles as parent advocates as a means to gain access to services in the health care and education system as well as in the community. In raising awareness, parents wanted to share their experiences and educate others as a means of promoting advocacy for parents of children and youth diagnosed with ASD to provide better outcomes for children in their life-long journey with ASD.

Our findings lend support to the work done by others. It is well recognized that parents of children with ASD and other chronic conditions or disabilities employ advocacy as a management or coping strategy that provides them with a sense of control over the various uncertainties faced.^32-34^ Parents identify barriers faced in their advocacy work including time commitments involved in parenting a child with ASD, financial challenges, lack of knowledge and support from service providers and others, lack of service availability and system bureaucracies, and perceived stigma related to their child’s ASD diagnosis that is consistent with previous findings.^32,33,35,36^ An online advocacy tool^37^ is available for parents of children with ASD, which includes information about the importance of teaching self-advocacy skills to parents and children with autism throughout the life course of the condition.^37^ The model of findings developed from this study provides an illustration to where these learned advocacy skills can be best used to achieve the desired best outcome for children with ASD. Understanding the advocacy process and steps to follow will help parents prepare and further develop their advocacy skills. Raising awareness about ASD, educating others, and supporting other parents in their journeys is important for parents that is echoed by others.^38^

Social psychology theories offer frameworks to inform advocacy and policy change efforts.^39,40^ For example, the “Grassroots” Theory of Change Model proposes that individuals affected by a problem act jointly to achieve social change using strategies such as mobilizing, training, developing awareness, and capacity-building.^40^ Results from this study demonstrate how parents can be actively involved and engaged in their advocacy efforts to raise public awareness of ASD. Service providers need to encourage parents to participate in advocacy training programs that are available as a means to achieve needed policy changes in the education and health systems.

Results from this study bring attention to the barriers parents face in their advocacy efforts and highlight the importance of reducing these challenges. Research in the U.S. studying advocacy training programs for parents of children with disabilities, including ASD, show significant gains in motivation, empowerment, and knowledge of special education^41,42^ and legislative rights.^42^ Another recent study aimed at understanding the impact of a volunteer advocacy project (VAP) on topics related to special education law and advocacy skills shows that families more likely to request an advocate if they live in an urban setting and have a child with ASD who attends elementary school.^7^ A recent pilot study of a special education advocacy program for Latinx-minority parents of children with ASD indicates that the program increased parental knowledge, but did not increase parents’ self-perceptions of empower and advocacy.^43^ This suggests that parents may need more support.^43^

We recognize the strengths and limitations to this study. This study reveals a thematic representation of parents’ advocacy journey. This new knowledge adds to our understanding of the advocacy skills parents must acquire when facing barriers to diagnosis and treatment for their children with ASD. Our sample for this qualitative study was restricted to parents living in Atlantic Canada and overrepresented highly educated, high-income mothers. These unique factors may limit the transferable of our populations.

To provide a 360° perspective on this issue, future research might explore the experiences of providers, policy makers, and adults with ASD to understand their perspectives on the strengths and limitations of the ASD service delivery system and explore strategies to promote parental advocacy. Future work may include conducting an intervention study focused on increasing parental advocacy to increase parents’ confidence in their ability to advocate on behalf of their children and to foster self-advocacy in adults through the life course of their condition.

## Implications for Practice

Results from this study raise several implications for practice. Although patient- and client-centered care is well recognized and widely accepted in health care settings, it is apparent from our findings that many parents of children with ASD do not experience this commitment to partnership. Parents contribute valuable information drawing on their understanding of their child and their observations of their child in their natural daily environment. This study highlights the importance of health care providers recognizing parents’ input and encouraging their advocacy efforts by including them in the decision-making process. A tailored approach for parents living in rural areas may address the additional geographical challenges related to accessing ASD services. Service providers are encouraged to link parents to appropriate ASD services early in the diagnosis process. Family physicians can refer parents to their local or provincial ASD advocacy group (eg, Autism Society of Newfoundland and Labrador) to provide information about ASD and assist parents to navigate the ASD delivery system (eg, information about provincial or local government programs and services that are available for families). There is a need for free community-based programs and services for parents, including respite care, self-care services, and parent counseling.

## Conclusions

The aim of this study was to gain a better understanding of the experience of advocacy in parents of children with ASD. Parental advocacy is a life-long process for parents of children with ASD. Advocacy is an ongoing effort where parents must continually anticipate their next course of action to acquire necessary supports for their children throughout the life course of their condition. Advocacy work includes providing a future to make things better for their child as they progress in their ASD journey. Parents are motivated through their advocacy efforts to support other parents navigating the process to avoid some barriers and have a more positive experience for them and their child.
